# PRELIMINARY EFFICACY OF AN OT INTERVENTION TO PREVENT FUNCTIONAL DECLINE AMONG OLDER ADULTS WITH CHRONIC DISEASES

**DOI:** 10.1093/geroni/igae098.4050

**Published:** 2024-12-31

**Authors:** Dalmina Arias, Mansha Mirza, Heidi Fischer

**Affiliations:** University of Illinois at Chicago, Chicago, Illinois, United States; University of Illinois Chicago, Chicago, Illinois, United States; University of Illinois Chicago, Chicago, Illinois, United States

## Abstract

Chronic disease affects 95% of older adults in the US, with 80% having two or more conditions. Poor management can lead to a decline in health, mobility, and ADL/IADL limitations, contributing to lower self-efficacy in disease management. Early identification and intervention of ADL/IADL limitations could help break the cycle of chronic disease and functional decline. This 2-arm parallel RCT explored the preliminary efficacy of an occupational therapy (OT) intervention for adults aged 55 and over with chronic conditions. The 12-week program included a comprehensive ADL assessment and ten intervention sessions focused on health management, physical deconditioning, and ADL/IADL limitations. Forty community-dwelling adults (mean age = 67 years) with diabetes or heart disease were recruited. Outcomes measured included physical functioning (Patient-Specific Functional Scale, Physical Performance Test, PROMIS Physical Functioning), self-efficacy for chronic disease management, health-related quality of life (PROMIS Global Health Measure), and ADL/IADL performance (Late-Life Functioning and Disability Index). ANCOVA was used for data analysis with baseline scores as covariates and Sidak correction for multiple comparisons. A significant effect favoring the intervention group was observed for the main outcome, the Patient-Specific Functional Scale (p =.04, d = 0.82, 95% CI [-1.5, -0.17]). Other outcomes showed promising trends with smaller effect sizes. These preliminary findings support the potential of OT interventions in managing chronic diseases among aging adults. Larger trials are needed to confirm these results.

